# Supplementary material for: Polyketide synthase gene clusters in the ericoid mycorrhizal fungus Oidiodendron maius: genome-wide prediction, expression profiling, and functional characterization of knockout mutants
Source: Front Plant Sci. 2026 Sep 16;17:1924430. doi: 10.3389/fpls.2026.1924430 (PMC13623903; doi:10.3389/fpls.2026.1924430)
Supplement: Supplementary file 1 [file SupplementaryFile1.zip › Supplementary Materials/Supplementary Folder 1.zip!/Oidma1_AssemblyScaffolds_Repeatmasked/knownclusterblast/cluster13/jgi.p_Oidma1_114650_mibig_hits.html]

| MIBiG Protein | Description | MIBiG Cluster | MiBiG Product | % ID | % Coverage | BLAST Score | E-value |
| --- | --- | --- | --- | --- | --- | --- | --- |
| CBF87076.1 | conserved\_hypothetical\_protein | BGC0001290 | Emericellamide\_A\_/\_emericellamide\_B\_biosynthetic\_gene\_cluster | 40 | 98.3022071307 | 405 | 8.9e-113 |
| AFT91380.1 | EcdI | BGC0000338 | Echinocandin\_B\_biosynthetic\_gene\_cluster | 38 | 97.7928692699 | 370 | 3.2e-102 |
| AMM63166.1 | AniI | BGC0001371 | Echinocandin\_B\_biosynthetic\_gene\_cluster | 38 | 97.7928692699 | 363 | 3.9e-100 |
| EPE34349.1 | Acetyl-CoA\_synthetase-like\_protein | BGC0001035 | Pneumocandin\_biosynthetic\_gene\_cluster | 37 | 97.7928692699 | 352 | 1.2e-96 |
| BAO73255.1 | AMP\_dependent\_ligase/synthetase | BGC0000456\_c4 | Verlamelin\_biosynthetic\_gene\_cluster | 31 | 99.6604414261 | 267 | 3.8e-71 |
| OAQ83766.1 | phenylacetyl-\_ligase\_protein | BGC0001358 | Leucinostatins\_biosynthetic\_gene\_cluster | 42 | 62.1392190153 | 253 | 4.3e-67 |
| EHA28233.1 | hypothetical\_protein | BGC0001143 | Azanigerone\_biosynthetic\_gene\_cluster | 33 | 81.494057725 | 179 | 1.4e-44 |
| BAH83502.1 | acyl-CoA\_synthetase | BGC0001253 | ACT-Toxin\_I\_biosynthetic\_gene\_cluster | 33 | 84.8896434635 | 173 | 5.7e-43 |
| AAX98210.1 | acyl\_CoA\_ligase | BGC0000052 | ECO-02301\_biosynthetic\_gene\_cluster | 27 | 91.3412563667 | 157 | 5.5e-38 |
| CAE14427.1 | long-chain-fatty-acid--CoA\_ligase\_(long-chain\_acyl-CoA\_synthetase) | BGC0001180\_c2 | Isopropylstilbene\_biosynthetic\_gene\_cluster | 30 | 81.324278438 | 156 | 9.4e-38 |
| ABJ90146.1 | MoeA4 | BGC0000805 | Moenomycin\_biosynthetic\_gene\_cluster | 29 | 84.8896434635 | 153 | 7.9e-37 |
| AGY30675.1 | Ann3 | BGC0001298 | 4-Z-annimycin\_biosynthetic\_gene\_cluster | 30 | 79.4567062818 | 151 | 3e-36 |
| ADI58647.1 | AsuD3 | BGC0000187 | Asukamycin\_biosynthetic\_gene\_cluster | 28 | 80.3056027165 | 150 | 5.2e-36 |
| ABS74201.1 | YngI | BGC0001095 | Fengycin\_biosynthetic\_gene\_cluster | 27 | 91.8505942275 | 146 | 7.4e-35 |
| CAE17553.1 | acyl\_CoA\_ligase | BGC0000210 | Chromomycin\_A3\_biosynthetic\_gene\_cluster | 31 | 61.6298811545 | 142 | 1.4e-33 |
| AAF08801.1 | YngI | BGC0001103 | Mycosubtilin\_biosynthetic\_gene\_cluster | 26 | 87.2665534805 | 134 | 2.9e-31 |
| AFU65902.1 | DacH | BGC0000216 | Dactylocycline\_biosynthetic\_gene\_cluster | 30 | 63.6672325976 | 134 | 5e-31 |
| AEW31026.1 | short-chain\_acyl-CoA\_synthetase | BGC0000407 | Plipastatin\_biosynthetic\_gene\_cluster | 25 | 86.7572156197 | 131 | 3.2e-30 |
| AEI98652.1 | CtcI | BGC0000209 | Chlortetracycline\_biosynthetic\_gene\_cluster | 28 | 77.2495755518 | 128 | 2.7e-29 |
| ABV97151.1 | AMP-dependent\_synthetase\_and\_ligase | BGC0000137 | Rifamycin\_biosynthetic\_gene\_cluster | 28 | 83.3616298812 | 126 | 1.4e-28 |
| AMY15066.1 | acyl-CoA\_synthetase/ligase | BGC0001339 | Squalestatin\_S1\_biosynthetic\_gene\_cluster | 25 | 89.9830220713 | 122 | 1.5e-27 |
| CAH10130.1 | feruloyl-CoA\_synthetase | BGC0000268 | Sch47554\_/\_Sch47555\_biosynthetic\_gene\_cluster | 27 | 82.3429541596 | 119 | 1.7e-26 |
| CAA07759.1 | acyl\_CoA\_ligase | BGC0000246 | Mithramycin\_biosynthetic\_gene\_cluster | 29 | 62.6485568761 | 113 | 9.1e-25 |
| CAK50779.1 | acyl\_CoA\_ligase | BGC0000247 | Mithramycin\_biosynthetic\_gene\_cluster | 29 | 62.6485568761 | 113 | 9.1e-25 |
| AHD25943.1 | ChdL | BGC0000208 | Chelocardin\_biosynthetic\_gene\_cluster | 29 | 64.0067911715 | 109 | 1.3e-23 |
| CQR60497.1 | Polyketide\_synthase,\_type\_I,\_modules:\_loading,\_1,\_2\_and\_3 | BGC0001287 | Chaxamycin\_A\_/\_chaxamycin\_B\_/\_chaxamycin\_C\_/\_chaxamycin\_D | 27 | 83.5314091681 | 106 | 1.1e-22 |
| CAO85890.1 | aryl-coA\_ligase | BGC0000110 | Neoaureothin\_biosynthetic\_gene\_cluster | 26 | 84.2105263158 | 106 | 1.5e-22 |
| ABC34346.1 | long-chain-fatty-acid--CoA\_ligase,\_putative | BGC0001102 | Malleilactone\_biosynthetic\_gene\_cluster | 26 | 85.9083191851 | 104 | 4.2e-22 |
| CBZ42143.1 | putative\_non-ribosomal\_peptide\_synthetase | BGC0001117 | Himastatin\_biosynthetic\_gene\_cluster | 28 | 64.5161290323 | 104 | 5.5e-22 |
| AAC01710.1 | RifA | BGC0000136 | Rifamycin\_biosynthetic\_gene\_cluster | 27 | 86.5874363328 | 102 | 1.2e-21 |
| AEW31022.1 | plipastatin\_synthetase | BGC0000407 | Plipastatin\_biosynthetic\_gene\_cluster | 28 | 66.2139219015 | 101 | 3.6e-21 |
| ACY06285.1 | non-ribosomal\_peptide\_synthetase | BGC0001042 | Sanglifehrin\_A\_biosynthetic\_gene\_cluster | 26 | 64.6859083192 | 99 | 1e-20 |
| AFV52199.1 | acyl-ACP\_synthetase | BGC0000081 | Kedarcidin\_biosynthetic\_gene\_cluster | 29 | 63.4974533107 | 97 | 5.2e-20 |
| AJV88376.1 | MfnD | BGC0001214 | Marformycins\_biosynthetic\_gene\_cluster | 28 | 56.1969439728 | 96 | 8.8e-20 |
| AEU11006.1 | NpnB | BGC0001029 | Nostophycin\_biosynthetic\_gene\_cluster | 24 | 85.7385398981 | 94 | 3.4e-19 |
| ABQ96384.2 | fusaricidin\_synthetase | BGC0001152 | Fusaricidin\_biosynthetic\_gene\_cluster | 28 | 59.9320882852 | 91 | 3.7e-18 |
| BAC67536.1 | arthrofactin\_synthetase\_C | BGC0000305 | Arthrofactin\_biosynthetic\_gene\_cluster | 24 | 90.3225806452 | 90 | 6.3e-18 |
| ADJ63842.1 | non-ribosomal\_peptide\_synthetase\_protein | BGC0000424 | Serobactins\_biosynthetic\_gene\_cluster | 29 | 71.8166383701 | 89 | 1.4e-17 |
| AAY37655.1 | Amino\_acid\_adenylation | BGC0000437 | Syringomycin\_biosynthetic\_gene\_cluster | 27 | 65.5348047538 | 89 | 1.4e-17 |
| AEH41794.1 | HrmP | BGC0000374 | Hormaomycin\_biosynthetic\_gene\_cluster | 25 | 66.0441426146 | 89 | 1.4e-17 |
| AHZ34243.1 | CipF | BGC0001389 | Cichopeptin\_biosynthetic\_gene\_cluster | 24 | 86.078098472 | 89 | 1.8e-17 |
| AAY37654.1 | Amino\_acid\_adenylation | BGC0000437 | Syringomycin\_biosynthetic\_gene\_cluster | 26 | 79.7962648557 | 89 | 1.8e-17 |
| EWS95124.1 | hypothetical\_protein | BGC0000306 | Arylomycin\_biosynthetic\_gene\_cluster | 24 | 84.3803056027 | 89 | 1.8e-17 |
| AAO72424.1 | syringopeptin\_synthetase\_B | BGC0000438 | Syringopeptin\_biosynthetic\_gene\_cluster | 26 | 79.7962648557 | 88 | 3.1e-17 |
| AAO72425.1 | syringopeptin\_synthetase\_C | BGC0000438 | Syringopeptin\_biosynthetic\_gene\_cluster | 27 | 65.5348047538 | 87 | 4.1e-17 |
| AAF99707.2 | syringopeptin\_synthetase | BGC0000438 | Syringopeptin\_biosynthetic\_gene\_cluster | 29 | 56.1969439728 | 86 | 9.1e-17 |
| CCM44336.1 | putative\_nonribosomal\_peptide\_synthetase | BGC0001056 | Zeamine\_biosynthetic\_gene\_cluster | 27 | 53.8200339559 | 86 | 9.1e-17 |
| WP\_010639240.1 | gramicidin\_synthetase | BGC0000958 | Antimycin\_biosynthetic\_gene\_cluster | 30 | 66.7232597623 | 86 | 1.2e-16 |
| AKA54626.1 | NRPS | BGC0001216 | Splenocin\_biosynthetic\_gene\_cluster | 29 | 66.5534804754 | 86 | 1.2e-16 |
| CBL93730.1 | NRPS | BGC0000360 | GE81112\_biosynthetic\_gene\_cluster | 27 | 65.7045840407 | 85 | 2e-16 |
| ABW00331.1 | amino\_acid\_adenylation\_domain | BGC0000333 | Cyclomarin\_biosynthetic\_gene\_cluster | 26 | 66.2139219015 | 85 | 2.7e-16 |
| AJV88377.1 | MfnE | BGC0001214 | Marformycins\_biosynthetic\_gene\_cluster | 26 | 58.5738539898 | 84 | 3.5e-16 |
| AAN32979.1 | BarE | BGC0000962 | Barbamide\_biosynthetic\_gene\_cluster | 28 | 51.4431239389 | 84 | 3.5e-16 |
| AAX31555.1 | acyl-CoA\_ligase | BGC0000336 | Daptomycin\_biosynthetic\_gene\_cluster | 29 | 59.5925297114 | 84 | 4.5e-16 |
| AAO62588.1 | peptide\_sythetase | BGC0001016 | Microcystin\_biosynthetic\_gene\_cluster | 24 | 92.3599320883 | 84 | 4.5e-16 |
| ACR33075.1 | proline\_adenylation\_protein | BGC0000017 | Anatoxin\_biosynthetic\_gene\_cluster | 24 | 65.3650254669 | 84 | 4.5e-16 |
| CAQ71826.1 | non\_ribosomal\_peptide\_synthase,\_antibiotic\_synthesis;\_contains\_1\_condensation\_domain,\_1\_AMP-acid\_ligases\_II\_domain,\_PP-binding,\_Phosphopantetheine\_attachment\_site\_and\_one\_taurine\_catabolism\_dioxygenase | BGC0001189 | Taiwachelin\_biosynthetic\_gene\_cluster | 27 | 58.9134125637 | 84 | 5.9e-16 |
| CDG17985.1 | Putative\_Ornithine\_racemase\_(fragment) | BGC0000464 | Xenoamicins\_biosynthetic\_gene\_cluster | 22 | 93.2088285229 | 84 | 5.9e-16 |
| AHZ34242.1 | CipE | BGC0001389 | Cichopeptin\_biosynthetic\_gene\_cluster | 25 | 71.3073005093 | 84 | 5.9e-16 |
| CAJ96471.1 | Non-ribosomal\_peptide\_synthetase | BGC0000330 | Cupriachelin\_biosynthetic\_gene\_cluster | 28 | 56.5365025467 | 83 | 7.7e-16 |
| ABD14711.1 | CesA | BGC0000320 | Cereulide\_biosynthetic\_gene\_cluster | 22 | 91.3412563667 | 83 | 7.7e-16 |
| BAH43871.1 | truncated\_linear\_pentadecapeptide\_gramicidin\_synthetase\_LgrC | BGC0000367 | Gramicidin\_biosynthetic\_gene\_cluster | 28 | 65.7045840407 | 82 | 1.3e-15 |
| AIG79241.1 | Hypothetical\_protein | BGC0000419 | Ristomycin\_A\_biosynthetic\_gene\_cluster | 26 | 68.5908319185 | 82 | 1.7e-15 |
| AKA59447.1 | non-ribosomal\_peptide\_synthetase | BGC0001203 | Clarexpoxcin\_biosynthetic\_gene\_cluster | 26 | 57.5551782683 | 82 | 2.3e-15 |
| BAF50711.1 | non\_ribosomal\_peptide\_synthetase\_for\_virginiamycin\_S | BGC0001116 | Virginiamycin\_biosynthetic\_gene\_cluster | 24 | 67.5721561969 | 82 | 2.3e-15 |
| ADZ24995.1 | non-ribosomal\_peptide\_synthase/polyketide\_synthase | BGC0000380 | Leupyrrin\_biosynthetic\_gene\_cluster | 26 | 70.9677419355 | 81 | 2.9e-15 |
| ALK27916.1 | non-ribosomal\_peptide\_synthase | BGC0001233 | Feglymycin\_biosynthetic\_gene\_cluster | 25 | 72.4957555178 | 81 | 3.8e-15 |
| CAA11795.1 | PCZA363.4 | BGC0000322 | Chloroeremomycin\_biosynthetic\_gene\_cluster | 26 | 70.2886247878 | 81 | 3.8e-15 |
| ADZ24989.1 | proline\_adenylation\_protein | BGC0000380 | Leupyrrin\_biosynthetic\_gene\_cluster | 26 | 65.7045840407 | 81 | 3.8e-15 |
| AAY93354.1 | non-ribosomal\_peptide\_synthetase\_PvdD | BGC0000413 | Pyoverdine\_biosynthetic\_gene\_cluster | 24 | 69.2699490662 | 81 | 3.8e-15 |
| AAY93355.1 | non-ribosomal\_peptide\_synthetase\_PvdJ | BGC0000413 | Pyoverdine\_biosynthetic\_gene\_cluster | 24 | 69.2699490662 | 81 | 3.8e-15 |
| CBJ79916.1 | putative\_Ornithine\_racemase | BGC0001133 | Taxlllaid\_biosynthetic\_gene\_cluster | 23 | 79.7962648557 | 81 | 5e-15 |
| CBJ79915.1 | putative\_Phenylalanine\_racemase\_(ATP-hydrolyzing) | BGC0001133 | Taxlllaid\_biosynthetic\_gene\_cluster | 23 | 79.7962648557 | 81 | 5e-15 |
| AGZ15459.1 | putative\_non-ribosomal\_peptide\_synthetase | BGC0001036 | Polyoxypeptin\_biosynthetic\_gene\_cluster | 24 | 92.1901528014 | 81 | 5e-15 |
| ADB12489.1 | EpoB | BGC0000990 | Epothilone\_biosynthetic\_gene\_cluster | 29 | 59.5925297114 | 80 | 6.5e-15 |
| ACA99172.1 | Polyketide\_synthase | BGC0001160 | Nonadecene\_biosynthetic\_gene\_cluster | 27 | 71.1375212224 | 80 | 6.5e-15 |
| ABF88003.1 | putative\_long-chain-fatty-acid\_CoA\_ligase | BGC0000871 | VEPE\_/\_AEPE\_/\_TG-1\_biosynthetic\_gene\_cluster | 24 | 76.740237691 | 80 | 8.6e-15 |
| ABD65956.1 | nonribosomal\_peptide\_synthetase | BGC0000341 | Enduracidin\_biosynthetic\_gene\_cluster | 29 | 53.8200339559 | 80 | 8.6e-15 |
| AAF62881.1 | EpoB | BGC0000991 | Epothilone\_biosynthetic\_gene\_cluster | 28 | 60.6112054329 | 79 | 1.1e-14 |
| BAC67534.2 | arthrofactin\_synthetase\_A | BGC0000305 | Arthrofactin\_biosynthetic\_gene\_cluster | 26 | 65.19524618 | 79 | 1.5e-14 |
| AGM14933.1 | xantholysin\_synthetase\_B | BGC0000463 | Xantholysin\_biosynthetic\_gene\_cluster | 28 | 52.4617996604 | 79 | 1.5e-14 |
| ADN26251.1 | ATP-dependent\_adenylase | BGC0000951 | Pacidamycin\_biosynthetic\_gene\_cluster | 25 | 69.9490662139 | 79 | 1.9e-14 |
| AEC14347.1 | nonribosomal\_peptide\_synthetase | BGC0000377 | Koranimine\_biosynthetic\_gene\_cluster | 28 | 54.3293718166 | 79 | 1.9e-14 |
| BAO84866.1 | putative\_non-ribosomal\_peptide\_synthetase | BGC0000414 | Quinocarcin\_biosynthetic\_gene\_cluster | 30 | 52.2920203735 | 79 | 1.9e-14 |
| AHB38497.1 | EpxD | BGC0000346 | Epoxomicin\_biosynthetic\_gene\_cluster | 26 | 57.7249575552 | 78 | 2.5e-14 |
| AHZ34238.1 | CipA | BGC0001389 | Cichopeptin\_biosynthetic\_gene\_cluster | 26 | 64.0067911715 | 78 | 2.5e-14 |
| KPN93064.1 | NupB | BGC0001416 | Nunapeptin\_/\_nunamycin\_biosynthetic\_gene\_cluster | 24 | 71.4770797963 | 78 | 3.2e-14 |
| BAP05590.1 | CalB | BGC0000967 | Calyculin\_biosynthetic\_gene\_cluster | 26 | 54.8387096774 | 78 | 3.2e-14 |
| AHZ34239.1 | CipB | BGC0001389 | Cichopeptin\_biosynthetic\_gene\_cluster | 26 | 64.0067911715 | 77 | 4.2e-14 |
| EOY45602.1 | non-ribosomal\_peptide\_synthetase | BGC0001168 | Livipeptin\_biosynthetic\_gene\_cluster | 29 | 54.4991511036 | 77 | 4.2e-14 |
| AEH59100.1 | amino\_acid\_adenylation\_domain-containing\_protein/NRPS | BGC0000385 | Lysobactin\_biosynthetic\_gene\_cluster | 26 | 82.0033955857 | 77 | 5.5e-14 |
| ADQ55476.1 | non-ribosomal\_peptide\_synthase\_dimodule | BGC0000350 | ET-743\_biosynthetic\_gene\_cluster | 24 | 57.5551782683 | 77 | 7.2e-14 |
| CAD17792.1 | probable\_non\_ribosomal\_peptide\_synthetase\_protein | BGC0001363 | Ralsolamycin\_biosynthetic\_gene\_cluster | 27 | 56.5365025467 | 77 | 7.2e-14 |
| AAF67501.2 | NovH | BGC0000834 | Novobiocin\_biosynthetic\_gene\_cluster | 27 | 66.3837011885 | 76 | 9.5e-14 |
| ACY13414.1 | amino\_acid\_adenylation\_domain\_protein | BGC0001367 | Haliamide\_biosynthetic\_gene\_cluster | 30 | 37.5212224109 | 76 | 9.5e-14 |
| AAP92491.1 | nonribosomal\_peptide\_synthetase | BGC0000458 | Viomycin\_biosynthetic\_gene\_cluster | 26 | 65.8743633277 | 76 | 9.5e-14 |
| CAB15186.3 | siderophore\_2,3-dihydroxybenzoate-glycine-threonine\_trimeric\_ester\_bacillibactin\_synthetase | BGC0000309 | Bacillibactin\_biosynthetic\_gene\_cluster | 25 | 66.0441426146 | 76 | 1.2e-13 |
| AAZ03550.1 | McnA | BGC0000332 | Cyanopeptolin\_biosynthetic\_gene\_cluster | 25 | 66.0441426146 | 76 | 1.2e-13 |
| AAF26925.1 | nonribosomal\_peptide\_synthetase | BGC0000988 | Epothilone\_biosynthetic\_gene\_cluster | 28 | 65.5348047538 | 76 | 1.6e-13 |
| ACB46193.1 | nonribosomal\_peptide\_synthetase | BGC0000989 | Epothilone\_biosynthetic\_gene\_cluster | 28 | 59.2529711375 | 76 | 1.6e-13 |
| ACM68690.1 | AerG1 | BGC0000298 | Aeruginosin\_biosynthetic\_gene\_cluster | 24 | 65.3650254669 | 75 | 2.1e-13 |
| BAH43766.1 | tyrocidine\_synthetase\_III | BGC0000452 | Tyrocidine\_biosynthetic\_gene\_cluster | 23 | 74.533106961 | 75 | 2.1e-13 |
| ADD82991.1 | PtnA2 | BGC0001156 | Platencin\_biosynthetic\_gene\_cluster | 26 | 62.1392190153 | 75 | 2.8e-13 |
| AHD05678.1 | nonribosomal\_peptide\_ligase\_subunit | BGC0000402 | Paenilarvins\_biosynthetic\_gene\_cluster | 26 | 64.1765704584 | 75 | 2.8e-13 |
| KPN93063.1 | NupA | BGC0001416 | Nunapeptin\_/\_nunamycin\_biosynthetic\_gene\_cluster | 25 | 65.19524618 | 75 | 2.8e-13 |
| ALD82525.1 | non-ribosomal\_peptide\_synthase | BGC0001212 | Nannocystin\_biosynthetic\_gene\_cluster | 26 | 54.6689303905 | 75 | 2.8e-13 |
| CAK15815.1 | putative\_non\_ribosomal\_peptide\_synthetase | BGC0000344 | Entolysin\_biosynthetic\_gene\_cluster | 25 | 72.6655348048 | 75 | 2.8e-13 |
| KPN90376.1 | NupC | BGC0001416 | Nunapeptin\_/\_nunamycin\_biosynthetic\_gene\_cluster | 24 | 90.4923599321 | 75 | 2.8e-13 |
| ALG65314.1 | Cal22 | BGC0001297 | WS9326\_biosynthetic\_gene\_cluster | 28 | 59.0831918506 | 74 | 3.6e-13 |
| AAU39359.1 | lichenysin\_synthase\_LchAA | BGC0000381 | Lichenysin\_biosynthetic\_gene\_cluster | 24 | 66.3837011885 | 74 | 4.7e-13 |
| AIW82280.1 | PuwC | BGC0001125 | Puwainaphycins\_biosynthetic\_gene\_cluster | 25 | 78.4380305603 | 74 | 4.7e-13 |
| ACZ65474.1 | palmitoyl-CoA\_synthetase | BGC0000140 | RK-682\_biosynthetic\_gene\_cluster | 29 | 56.3667232598 | 74 | 6.1e-13 |
| ABS74206.1 | FenD | BGC0001095 | Fengycin\_biosynthetic\_gene\_cluster | 25 | 67.7419354839 | 74 | 6.1e-13 |
| CDG17982.1 | Non-ribosomal\_peptide\_synthetase | BGC0000464 | Xenoamicins\_biosynthetic\_gene\_cluster | 22 | 94.0577249576 | 73 | 8e-13 |
| AJM89735.1 | PmxA | BGC0001192 | Colistin\_biosynthetic\_gene\_cluster | 26 | 67.2325976231 | 73 | 1e-12 |
| AFH75329.1 | nonribosomal\_peptide\_synthetase | BGC0000398 | Orfamide\_biosynthetic\_gene\_cluster | 23 | 70.9677419355 | 72 | 1.4e-12 |
| BAB69698.1 | iturin\_A\_synthetase\_A | BGC0001098 | Iturin\_biosynthetic\_gene\_cluster | 24 | 72.156196944 | 72 | 1.8e-12 |
| CBD77754.1 | hypothetical\_protein | BGC0000974 | Crocacin\_biosynthetic\_gene\_cluster | 23 | 73.3446519525 | 72 | 1.8e-12 |
| ABW17376.1 | PsoB | BGC0000411 | Putisolvin\_biosynthetic\_gene\_cluster | 26 | 68.5908319185 | 72 | 1.8e-12 |
| AFJ23825.1 | WLIP\_synthetase\_B | BGC0000462\_c2 | WLIP\_biosynthetic\_gene\_cluster | 26 | 67.5721561969 | 72 | 1.8e-12 |
| CAK15814.1 | putative\_non-ribosomal\_peptide\_synthetase,\_terminal\_component | BGC0000344 | Entolysin\_biosynthetic\_gene\_cluster | 27 | 68.7606112054 | 72 | 2.3e-12 |
| ACU71638.1 | amino\_acid\_adenylation\_domain\_protein | BGC0001154 | Cacibiocin\_biosynthetic\_gene\_cluster | 26 | 66.8930390492 | 72 | 2.3e-12 |
| ABS74180.1 | BmyB | BGC0001090 | Bacillomycin\_biosynthetic\_gene\_cluster | 25 | 65.7045840407 | 71 | 3e-12 |
| AAY93445.1 | non-ribosomal\_peptide\_synthetase\_PvdL | BGC0000413 | Pyoverdine\_biosynthetic\_gene\_cluster | 26 | 67.40237691 | 71 | 3e-12 |
| AAT09804.1 | NocA | BGC0000395 | Nocardicin\_A\_biosynthetic\_gene\_cluster | 25 | 73.0050933786 | 71 | 4e-12 |
| AAY37653.1 | Amino\_acid\_adenylation | BGC0000437 | Syringomycin\_biosynthetic\_gene\_cluster | 26 | 53.1409168081 | 71 | 4e-12 |
| CAA16183.1 | polyketide\_synthase | BGC0001063 | Undecylprodigiosin\_biosynthetic\_gene\_cluster | 24 | 60.6112054329 | 71 | 5.2e-12 |
| CAO98843.1 | hypothetical\_protein | BGC0000023 | Aurafuron\_biosynthetic\_gene\_cluster | 27 | 73.1748726655 | 71 | 5.2e-12 |
| AHD05679.1 | putative\_non-ribosomal\_peptide\_ligase/\_polyketide\_synthase\_hybrid | BGC0000402 | Paenilarvins\_biosynthetic\_gene\_cluster | 24 | 73.0050933786 | 71 | 5.2e-12 |
| BAG84247.1 | putative\_L-prolyl-AMP\_ligase | BGC0000257 | Prodigiosin\_biosynthetic\_gene\_cluster | 26 | 55.857385399 | 70 | 6.8e-12 |
| ADN13832.1 | Beta-ketoacyl\_synthase | BGC0001164 | 1-heptadecene\_biosynthetic\_gene\_cluster | 28 | 57.3853989813 | 70 | 6.8e-12 |
| AAY91420.2 | non-ribosomal\_peptide\_synthetase\_OfaB | BGC0000399 | Orfamide\_biosynthetic\_gene\_cluster | 23 | 72.3259762309 | 70 | 8.8e-12 |
| ABS74181.1 | BmyA | BGC0001090 | Bacillomycin\_biosynthetic\_gene\_cluster | 24 | 72.3259762309 | 69 | 1.5e-11 |
| KPN93065.1 | NunD | BGC0001416 | Nunapeptin\_/\_nunamycin\_biosynthetic\_gene\_cluster | 24 | 66.5534804754 | 69 | 2e-11 |
| CAD29795.1 | peptide\_synthetase | BGC0001015 | Microcystin\_biosynthetic\_gene\_cluster | 24 | 90.3225806452 | 69 | 2e-11 |
| CAQ43084.1 | non\_ribosomal\_polypeptide\_synthetase | BGC0000970 | Chondrochloren\_biosynthetic\_gene\_cluster | 24 | 68.0814940577 | 68 | 2.6e-11 |
| AGD80618.1 | non-ribosomal\_peptide\_synthetase | BGC0000394 | Naphthyridinomycin\_biosynthetic\_gene\_cluster | 27 | 54.6689303905 | 68 | 2.6e-11 |
| AFV27434.1 | indigoidine\_synthase | BGC0000375 | Indigoidine\_biosynthetic\_gene\_cluster | 25 | 57.3853989813 | 68 | 2.6e-11 |
| ABV56585.1 | KtzE | BGC0000378 | Kutznerides\_biosynthetic\_gene\_cluster | 25 | 60.441426146 | 68 | 3.4e-11 |
| AHF22854.1 | MarL | BGC0000091 | Marineosin\_biosynthetic\_gene\_cluster | 26 | 57.2156196944 | 67 | 4.4e-11 |
| CBF87069.1 | nonribosomal\_peptide\_synthase,\_putative\_(Eurofung) | BGC0001290 | Emericellamide\_A\_/\_emericellamide\_B\_biosynthetic\_gene\_cluster | 24 | 69.2699490662 | 67 | 5.7e-11 |
| AAY37647.1 | Amino\_acid\_adenylation | BGC0000437 | Syringomycin\_biosynthetic\_gene\_cluster | 26 | 71.1375212224 | 67 | 7.5e-11 |
| AGE25479.1 | putative\_non-ribosomal\_peptide\_synthetase | BGC0001208\_c2 | Poaeamide\_biosynthetic\_gene\_cluster | 26 | 56.1969439728 | 66 | 9.8e-11 |
| AAO62585.1 | peptide\_sythetase\_polyketide\_synthase\_fusion\_protein | BGC0001016 | Microcystin\_biosynthetic\_gene\_cluster | 25 | 73.5144312394 | 66 | 1.3e-10 |
| ACK77757.1 | putative\_indigoidine\_synthase\_IndC | BGC0000727 | Auricin\_deoxysugar\_moieties\_biosynthetic\_gene\_cluster | 22 | 77.4193548387 | 66 | 1.7e-10 |
| AEZ64574.1 | fatty-acid-CoA\_ligase | BGC0001065 | Herboxidiene\_biosynthetic\_gene\_cluster | 22 | 75.7215619694 | 66 | 1.7e-10 |
| CAL80821.1 | sylD-like\_NRPS/PKS | BGC0000997 | Glidobactin\_biosynthetic\_gene\_cluster | 23 | 65.19524618 | 65 | 2.2e-10 |
| AAF08795.1 | MycA | BGC0001103 | Mycosubtilin\_biosynthetic\_gene\_cluster | 24 | 70.2886247878 | 65 | 2.2e-10 |
| ABX37385.1 | amino\_acid\_adenylation\_domain\_protein | BGC0000984 | Delftibactin\_biosynthetic\_gene\_cluster | 27 | 54.4991511036 | 65 | 2.2e-10 |
| AAO56329.1 | non-ribosomal\_peptide\_synthetase\_SyfB | BGC0000435 | Syringafactin\_biosynthetic\_gene\_cluster | 25 | 54.6689303905 | 65 | 2.8e-10 |
| AFK57215.1 | DidD | BGC0000985 | Didemnin\_biosynthetic\_gene\_cluster | 27 | 40.4074702886 | 65 | 2.8e-10 |
| ABL74940.1 | TlmVI | BGC0001048 | Tallysomycin\_biosynthetic\_gene\_cluster | 24 | 79.4567062818 | 65 | 2.8e-10 |
| AAU34202.1 | mannopeptimycin\_peptide\_synthetase\_MppA | BGC0000388 | Mannopeptimycin\_biosynthetic\_gene\_cluster | 26 | 59.9320882852 | 65 | 2.8e-10 |
| ABF87031.1 | non-ribosomal\_peptide\_synthetase/polyketide\_synthase | BGC0000393 | Myxoprincomide\_biosynthetic\_gene\_cluster | 25 | 61.7996604414 | 64 | 3.7e-10 |
| AEH59099.1 | amino\_acid\_adenylation\_domain-containing\_protein/NRPS | BGC0000385 | Lysobactin\_biosynthetic\_gene\_cluster | 26 | 72.6655348048 | 64 | 6.3e-10 |
| CAE14173.1 |  | BGC0000383 | Luminmycin\_biosynthetic\_gene\_cluster | 23 | 66.0441426146 | 64 | 6.3e-10 |
| ABD14712.1 | CesB | BGC0000320 | Cereulide\_biosynthetic\_gene\_cluster | 22 | 63.6672325976 | 63 | 8.3e-10 |
| CDF96614.1 | Long-chain-fatty-acid--CoA\_ligase | BGC0001149 | Brabantamide\_biosynthetic\_gene\_cluster | 23 | 76.740237691 | 63 | 8.3e-10 |
| ABW17377.1 | PsoC | BGC0000411 | Putisolvin\_biosynthetic\_gene\_cluster | 27 | 68.0814940577 | 63 | 8.3e-10 |
| AJI44176.1 | nonribosomal\_peptide\_synthetase | BGC0001193 | Thiolutin\_biosynthetic\_gene\_cluster | 24 | 64.0067911715 | 62 | 1.4e-09 |
| AGN74885.1 | nonribosomal\_peptide\_synthetase | BGC0000459 | Griseoviridin\_/\_viridogrisein\_biosynthetic\_gene\_cluster | 22 | 93.3786078098 | 62 | 1.4e-09 |
| AAK81826.1 | peptide\_synthetase | BGC0000326 | Complestatin\_biosynthetic\_gene\_cluster | 27 | 56.1969439728 | 62 | 1.8e-09 |
| ALV86864.1 | Tlo18 | BGC0001406 | Telomycin\_biosynthetic\_gene\_cluster | 25 | 70.7979626486 | 62 | 1.8e-09 |
| AFV96135.1 | AMP-dependent\_synthetase/ligase | BGC0001064 | Cylindrocyclophane\_biosynthetic\_gene\_cluster | 26 | 56.5365025467 | 62 | 1.8e-09 |
| AHB82069.1 | non\_ribosomal\_peptide\_synthetase | BGC0001231 | Microsclerodermins\_biosynthetic\_gene\_cluster | 27 | 53.480475382 | 62 | 2.4e-09 |
| ADD82940.1 | Bat2 | BGC0001099 | Kalimantacin\_/\_batumin\_biosynthetic\_gene\_cluster | 23 | 64.5161290323 | 62 | 2.4e-09 |
| AHF22853.1 | MarM | BGC0000091 | Marineosin\_biosynthetic\_gene\_cluster | 25 | 51.6129032258 | 62 | 2.4e-09 |
| CDG17987.1 | Putative\_Ornithine\_racemase\_(fragment) | BGC0000464 | Xenoamicins\_biosynthetic\_gene\_cluster | 26 | 70.4584040747 | 61 | 3.1e-09 |
| BAC67535.1 | arthrofactin\_synthetase\_B | BGC0000305 | Arthrofactin\_biosynthetic\_gene\_cluster | 23 | 82.5127334465 | 61 | 4.1e-09 |
| AJV88375.1 | MfnC | BGC0001214 | Marformycins\_biosynthetic\_gene\_cluster | 28 | 53.1409168081 | 61 | 4.1e-09 |
| AAY89051.1 | hybrid\_nonribosomal\_peptide\_synthetase/polyketide\_synthase | BGC0001069 | Chivosazole\_biosynthetic\_gene\_cluster | 27 | 55.1782682513 | 61 | 4.1e-09 |
| ALV86868.1 | Tlo22 | BGC0001406 | Telomycin\_biosynthetic\_gene\_cluster | 23 | 64.8556876061 | 61 | 4.1e-09 |
| AAN85512.1 | nonribosomal\_peptide\_synthetase | BGC0001101 | Leinamycin\_biosynthetic\_gene\_cluster | 25 | 72.6655348048 | 61 | 5.4e-09 |
| AFH75322.1 | nonribosomal\_peptide\_synthetase | BGC0000425 | Sessilin\_biosynthetic\_gene\_cluster | 23 | 73.8539898132 | 61 | 5.4e-09 |
| ADM34138.1 | non-ribosomal\_peptide\_synthetase | BGC0001084 | Notoamide\_/\_stephacidin\_biosynthetic\_gene\_cluster | 25 | 54.4991511036 | 61 | 5.4e-09 |
| AAN32981.1 | BarG | BGC0000962 | Barbamide\_biosynthetic\_gene\_cluster | 24 | 55.5178268251 | 60 | 7e-09 |
| AHD05621.1 | non-ribosomal\_peptide\_ligase\_domain\_protein | BGC0001033 | Paenilamicin\_biosynthetic\_gene\_cluster | 22 | 64.6859083192 | 60 | 9.2e-09 |
| AFH75320.1 | nonribosomal\_peptide\_synthetase | BGC0000425 | Sessilin\_biosynthetic\_gene\_cluster | 24 | 70.4584040747 | 59 | 1.2e-08 |
| AHH34188.1 | fatty\_acyl\_ACP\_ligase | BGC0001162 | 1-heptadecene\_biosynthetic\_gene\_cluster | 30 | 30.5602716469 | 59 | 1.2e-08 |
| ALK27915.1 | non-ribosomal\_peptide\_synthase | BGC0001233 | Feglymycin\_biosynthetic\_gene\_cluster | 22 | 93.5483870968 | 59 | 1.2e-08 |
| EGJ35087.1 | AMP-forming\_acyl-CoA\_synthetase | BGC0001163 | 1-heptadecene\_biosynthetic\_gene\_cluster | 30 | 30.5602716469 | 59 | 1.6e-08 |
| CBW45640.1 | PII\_peptide\_synthetase | BGC0000952 | Pristinamycin\_biosynthetic\_gene\_cluster | 26 | 56.7062818336 | 59 | 1.6e-08 |
| CCJ67636.1 | TaaA | BGC0000447 | Tolaasin\_biosynthetic\_gene\_cluster | 23 | 70.4584040747 | 59 | 2e-08 |
| CAB38517.1 | CDA\_peptide\_synthetase\_II | BGC0000315 | Calcium-dependent\_antibiotic\_biosynthetic\_gene\_cluster | 25 | 64.5161290323 | 58 | 3.5e-08 |
| AGD80622.1 | AMP-dependent\_synthetase\_and\_ligase | BGC0000394 | Naphthyridinomycin\_biosynthetic\_gene\_cluster | 26 | 50.0848896435 | 58 | 3.5e-08 |
| AOA33122.1 | nonribosomal\_peptide\_synthetase | BGC0001346 | Bananamides\_biosynthetic\_gene\_cluster | 24 | 67.0628183362 | 58 | 3.5e-08 |
| AED90002.1 | non-ribosomal\_peptide\_synthetase\_ThaA | BGC0000443 | Thanamycin\_biosynthetic\_gene\_cluster | 25 | 70.6281833616 | 57 | 4.5e-08 |
| CCJ67640.1 | TaaE | BGC0000447 | Tolaasin\_biosynthetic\_gene\_cluster | 24 | 58.234295416 | 57 | 4.5e-08 |
| CCA89328.1 | mixed\_trans-AT\_type\_I\_polyketide\_synthase/nonribosomal\_peptide\_synthetase | BGC0001111 | Rhizopodins\_biosynthetic\_gene\_cluster | 25 | 57.8947368421 | 57 | 4.5e-08 |
| CAM59606.1 | non-ribosomal\_peptide\_synthetase | BGC0000297 | Aeruginoside\_biosynthetic\_gene\_cluster | 25 | 54.1595925297 | 57 | 5.9e-08 |
| CAJ76292.1 | putative\_non-ribosomal\_peptide\_synthase | BGC0000972 | Colibactin\_biosynthetic\_gene\_cluster | 25 | 51.6129032258 | 57 | 7.8e-08 |
| CDG17980.1 | Putative\_Ornithine\_racemase\_(fragment) | BGC0000464 | Xenoamicins\_biosynthetic\_gene\_cluster | 24 | 90.4923599321 | 56 | 1e-07 |
| CBG75492.1 | putative\_NRPS/siderophore\_biosynthesis\_protein | BGC0000423 | Scabichelin\_biosynthetic\_gene\_cluster | 29 | 33.1069609508 | 56 | 1.3e-07 |
| AHH34187.1 | fatty\_acyl\_ACP\_ligase | BGC0001161 | 1-heptadecene\_biosynthetic\_gene\_cluster | 30 | 29.5415959253 | 56 | 1.3e-07 |
| ALK27914.1 | non-ribosomal\_peptide\_synthase | BGC0001233 | Feglymycin\_biosynthetic\_gene\_cluster | 25 | 55.6876061121 | 55 | 2.3e-07 |
| AKP45396.1 | CysH | BGC0001413 | Cystobactamide\_biosynthetic\_gene\_cluster | 26 | 52.6315789474 | 54 | 3.8e-07 |
| CRG85572.1 | nonribosomal\_peptide\_synthase,\_putative | BGC0001402 | Cyclochlorotine\_biosynthetic\_gene\_cluster | 24 | 45.3310696095 | 54 | 6.6e-07 |
| CAQ18831.1 | nonribosomal\_polypetide\_synthetase | BGC0000954 | Ajudazol\_biosynthetic\_gene\_cluster | 25 | 53.8200339559 | 53 | 8.6e-07 |
| AMK48227.1 | nonribosomal\_peptide\_synthetas | BGC0001351 | Cahuitamycins\_biosynthetic\_gene\_cluster | 33 | 20.7130730051 | 53 | 1.1e-06 |
| AAY91421.3 | non-ribosomal\_peptide\_synthetase\_OfaC | BGC0000399 | Orfamide\_biosynthetic\_gene\_cluster | 24 | 37.3514431239 | 52 | 1.5e-06 |
| AAG02359.1 | peptide\_synthetase\_NRPS5-4-3 | BGC0000963 | Bleomycin\_biosynthetic\_gene\_cluster | 28 | 31.9185059423 | 52 | 1.9e-06 |
| AAW49319.1 | thaxtomin\_synthetase\_B | BGC0000444 | Thaxtomin\_biosynthetic\_gene\_cluster | 31 | 24.2784380306 | 52 | 1.9e-06 |
| AGE25480.1 | amino\_acid\_adenylation\_protein | BGC0001208\_c2 | Poaeamide\_biosynthetic\_gene\_cluster | 28 | 52.4617996604 | 52 | 2.5e-06 |
| EFJ68969.1 | AMP-binding\_enzyme | BGC0000628 | Lactocillin\_biosynthetic\_gene\_cluster | 24 | 32.7674023769 | 50 | 5.6e-06 |
